# Acetate Kinase Isozymes Confer Robustness in Acetate Metabolism

**DOI:** 10.1371/journal.pone.0092256

**Published:** 2014-03-17

**Authors:** Siu Hung Joshua Chan, Lasse Nørregaard, Christian Solem, Peter Ruhdal Jensen

**Affiliations:** National Food Institute, Technical University of Denmark, Kgs. Lyngby, Denmark; Oak Ridge National Laboratory, United States of America

## Abstract

Acetate kinase (ACK) (EC no: 2.7.2.1) interconverts acetyl-phosphate and acetate to either catabolize or synthesize acetyl-CoA dependent on the metabolic requirement. Among all ACK entries available in UniProt, we found that around 45% are multiple ACKs in some organisms including more than 300 species but surprisingly, little work has been done to clarify whether this has any significance. In an attempt to gain further insight we have studied the two ACKs (AckA1, AckA2) encoded by two neighboring genes conserved in *Lactococcus lactis* (*L. lactis*) by analyzing protein sequences, characterizing transcription structure, determining enzyme characteristics and effect on growth physiology. The results show that the two ACKs are most likely individually transcribed. AckA1 has a much higher turnover number and AckA2 has a much higher affinity for acetate *in vitro*. Consistently, growth experiments of mutant strains reveal that AckA1 has a higher capacity for acetate production which allows faster growth in an environment with high acetate concentration. Meanwhile, AckA2 is important for fast acetate-dependent growth at low concentration of acetate. The results demonstrate that the two ACKs have complementary physiological roles in *L. lactis* to maintain a robust acetate metabolism for fast growth at different extracellular acetate concentrations. The existence of ACK isozymes may reflect a common evolutionary strategy in bacteria in an environment with varying concentrations of acetate.

## Introduction

There are many examples where products of metabolism are excreted and later re-assimilated by organisms, e.g. acetate (OAc) in *E. coli* [1] and ethanol in yeast [2]. The ability to switch between dissimilation and assimilation of the same metabolite is an important trait for maximizing growth in a changing environment. The acetate switch is a prominent example of this type of behavior. Acetate is one of the main metabolic products, and re-assimilation happens in order to exploit an available carbon source for further biomass formation after the primary carbon source has been depleted. A general review of the switch in acetate metabolism can be found in Wolfe [3].

**Figure 1:**
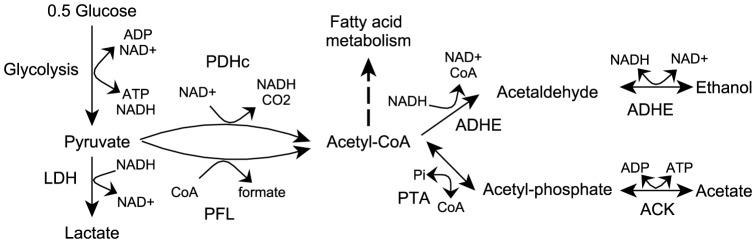
Pyruvate and Ac-CoA metabolism. Pi: inorganic phosphate. CO_2_: carbon dioxide. Enzyme names are in bold. LDH: lactate dehydrogenase. PFL: pyruvate formate lyase. PHDc: pyruvate dehydrogenase complex. PTA: phosphotransacetylase. ACK: acetate kinase. ADHE: bi-functional alcohol dehydrogenase.

*Lactococcus lactis* (*L. lactis*) is an important Gram-positive model organism which belongs to the group of Lactic Acid Bacteria (LAB) and is widely used in cheese production. In *L. lactis*, acetate may also be excreted or assimilated, depending on the environmental conditions. Fig.1 summarizes the relevant metabolic reactions. Under anaerobic conditions *L. lactis* produces mainly lactate as well as formate, ethanol and acetate. The amounts of formate, ethanol and acetate become very significant when growth depends on slowly fermentable sugars like maltose and galactose [4]. Acetate can also be a precursor of Ac-CoA which is vital to *L. lactis* because Ac-CoA is a precursor in fatty acid biosynthesis [5], cysteine biosynthesis [6] and peptidoglycan biosynthesis [7], etc. Acetate is required for growth when other routes to Ac-CoA are blocked. In *L. lactis* there are three known pathways leading to Ac-CoA, including the pyruvate dehydrogenase complex (PDHc), pyruvate formate lyase (PFL) and phosphotransacetylase (PTA) in conjunction with acetate kinase (ACK) (Fig. 1). PDHc is mainly active under aerobic conditions in the presence of the cofactor lipoic acid. Under strict anaerobiosis, PDHc was shown to have low activity [8] and growth depends on PFL in the absence of acetate [9], [10]. PFL is active only anaerobically due to inactivation by oxygen [11]. The PTA-ACK pathway, which converts acetate into Ac-CoA, can support growth when PDHc and PFL are both inactive provided that acetate is added to the media. If all the three known pathways leading to Ac-CoA are blocked, *L. lactis* is unable to grow [9]. A gene predicted to encode either the AMP-forming Ac-CoA synthetase (ACS) (which is important for Ac-CoA production from acetate in *E. coli* [3]) or acyl-CoA synthetase is also present in some *L. lactis* strains but no ACS activity has been reported. There are no other annotated genes in *L. lactis* encoding enzymes known to catalyze the conversion between Ac-CoA and acetate.

Interestingly, in all available sequenced genomes of *L. lactis*, two well conserved neighboring homologous genes are predicted to encode ACK. A further search on all available ACK sequences shows that in fact >300 species have multiple ACKs, including some other LAB species. The prevalence suggests a plausible advantage conferred by multiple ACK genes to *L. lactis* and other bacteria. In the literature, the kinetics and mechanism of ACK have long been a subject of study. The kinetics of ACK among different organisms has been characterized, e.g. [12]–[15]. Crystallographic studies, site-specific mutagenesis and sequence comparisons have revealed sites important for substrate binding and catalysis, e.g. [13], [16]–[19]. Several reaction mechanisms have also been proposed [20]–[22]. Despite the massive amount of literature on ACK, surprisingly, little work has been done on ACK isozymes which exist in many species, with the exception of one kinetic study in a spirochete [23].

In the hope of revealing the significance of ACK isozymes, in this study we investigated the two ACKs in *L. lactis* by sequence analysis, characterization of transcription structure, enzyme activity and effect on growth physiology.

## Materials and Methods

### Bacterial Strains and Plasmids

All the *L. lactis* strains involved in this study were derived from the plasmid-free laboratory strain *L. lactis subsp. cremoris* MG1363 [24]. For overexpression of ACKs, *E. coli strain* M15 pREP4 groESL [25] was used. The plasmid pCS1966 containing genes encoding erythromycin resistance and an orotate transporter was used for markerless gene inactivation in *L. lactis* [26]. The plasmids pLB65, harboring a gene encoding a site-specific integrase, and pLB85, containing the *gusA* reporter gene and a gene encoding erythromycin resistance, were used for constructing strains needed for *in vivo* promoter strength assessment [27]. The primers, plasmids and strains used in the study are listed in Table S1, Table S2 and Table S3 respectively.

### Antibiotics

When needed erythromycin was added at 5 μg ml^−1^ for *L. lactis*. Ampicillin and kanamycin were applied at 100 μg ml^−1^ and 25 μg ml^−1^ respectively for *E. coli*.

### Culture Media and Growth Conditions

*E. coli* was grown aerobically at 37°C in Lysogeny Broth (LB). *L. lactis* strains were cultivated at 30°C without aeration in M17 broth supplemented with 2 g L^−1^ of glucose or in chemically defined SA medium [28] devoid of acetate and supplemented with 2 g L^−1^ of maltose (MSA). *L. lactis* growth experiments were carried out in flasks at 30°C under static conditions with slow stirring and optical density at 600 nm (OD_600_) was measured regularly. As inoculum an over-night exponentially growing culture in the same medium was used and the start OD_600_≈0.02. The growth rate was calculated as the average of three replications. The cell density was correlated to the cell mass of *L. lactis* to be 0.36 g (dry weight) per liter of SA medium of OD_600_ = 1.

### Quantification of Maltose and Fermentation Products

HPLC was employed to measure the concentration of maltose, lactate, formate and acetate in the samples taken during the growth experiments as previously described [29].

### DNA Techniques

The method used to isolate the chromosomal DNA from *L. lactis* was modified from a previous method [30]. PCR amplification, restriction, ligation, transformation and plasmid purification from *E. coli* were performed following procedures described in Sambrook et al. [31] and the description from the manufacturer of the enzymes used. Electrocompetent cells of *L. lactis* were grown in M17 broth supplemented with 10 g L^−1^ glucose and 10 g L^−1^ glycine and transformed by electroporation as described previously [32].

### Gene Inactivation

Gene inactivation was achieved by deleting the whole gene or part of the gene containing the necessary active sites using the plasmid pCS1966 [26]. ≈800-bp regions upstream and downstream of the target to be deleted were PCR amplified and inserted into pCS1966. The resulting plasmids were used as previously described [26].

### Construction of *gusA* Reporter Strains

The promoter containing region upstream a specific gene was PCR amplified and inserted into plasmid pLB85 and transformed into the desired *L. lactis* strain expressing phage TP901-1 integrase as described previously [27]. Transformants were selected on GM17 with erythromycin and verified by sequencing using primers CSO50 and CSO263 (Table S1).

### Rapid Amplification of cDNA Ends (RACE)

For RNA isolation, cells of MG1363 were harvested from an exponentially growing SA culture supplemented with 2 g L^−1^ glucose or maltose, with 2 μg ml^−1^ of lipoic acid and nucleosides. Cells were then resuspended in 200 μl Solution I (0.3 M sucrose and 0.01 M NaAc, pH 4.8) and 200 μl preheated Solution II (2% SDS and 0.01 M NaAc, pH 4.8). 400 μl phenol/acetate (phenol equilibrated with 100 mM NaAc, pH 4.8) was added and the mixture was disrupted by glass beads (106-μm diameter; Sigma, Prod. No. G4649) using a FastPrep (MP Biomedicals, Santa Ana, USA). The resulting lysate was centrifuged and the water phase was extracted by phenol/acetate two times and finally by phenol/acetate mixed with chloroform in a 1∶1 ratio. RNA was precipitated by ethanol and dissolved in DEPC-treated water. RACE was performed using the SMARTer™ RACE cDNA Amplification Kit (Clontech) according to the instructions of the manufacturer.

### Overproduction of *L. lactis* ACK in *E. coli*

The two ACK genes (*ackA1*, *ackA2*) from MG1363 were PCR amplified using primers 71f, 71r and 62f, 62r respectively. After digestion with BglII, SalI and BamHI, SalI respectively, the fragments were inserted into the vector pQE30 (Qiagen) digested with the same enzymes and subsequently introduced into the *E. coli* strain M15 pREP4*groESL* [25]. The strains were grown and His-tagged ACKs were produced via IPTG induction and purification on a Ni-NTA resin (Qiagen) according to the manufacturer's instruction. Purified protein was gel-filtrated on a PD-10 column (GE Healthcare) thereby transferring it to Solution A (50 mM Tris-HCl pH 7.5, 100 mM NaCl, 10% glycerol). Protein concentration was determined using Bradford Reagent (Sigma, Prod. No. B6916) and a protein standard (200 mg ml^−1^ BSA, Sigma, Prod. No. P5369), following the protocol provided by the manufacturer. The molecular weight of the protein was estimated by gel filtration using a HiPrep™ 16/60 Sephacryl™ S-300 High Resolution column (GE Healthcare) and a Gel Filtration Standard (BioRad, Cat. No. 151-1901). The mobile phase used was 0.05 M sodium phosphate, 0.15 M NaCl, pH 7.0 and the flow rate was 0.2 ml min^−1^. Proteins were detected using the Ultimate 3000 Diode Array Detector (Dionex) at 280 nm.

### Measurement of ACK Activities

ACK activities were measured on either purified proteins or in cell extracts. Cell extracts were obtained by harvesting exponentially growing cells which were then resuspended in extract buffer [29] and disrupted by glass beads (106-μm diameter; Sigma, Prod. No. G4649) using a FastPrep (MP Biomedicals, Santa Ana, USA). The master buffer used for the assay was adapted from Goel et al. [33]: 100 mM HEPES, 50 mM NaCl, 400 mM potassium glutamate, 1 mM potassium phosphate and 10× diluted metal ions present in SA medium, adjusted to pH 7.5 with potassium hydroxide. For the production of acetate from Ac-P, the same assay mix was used as in Goel et al. [33]: master buffer, 5 mM MgSO_4_, 2 mM D-glucose, 0.4 mM NAD^+^, 8.5 U ml^−1^ hexokinase, 12.7 U ml^−1^ D-glucose 6-phosphate dehydrogenase, with varying amounts of ADP and Ac-P. For measurements of V_max_ in cell extracts, 3 mM ADP and 2 mM Ac-P were used. For the reverse direction, the assay mix was modified from a previous article [34]: master buffer, 4.2 mM MgCl_2_, 1.7 mM phosphoenolpyruvate, 0.24 mM NADH, 9 U ml^−1^ pyruvate kinase, 12 U ml^−1^ lactate dehydrogenase, with varying amounts of ATP and potassium acetate. For measurement of V_max_ in cell extracts, 4 mM ATP and 200 mM acetate were used. The enzyme activities were determined by monitoring OD_340_ corresponding to the concentration of NADH using the Infinite® M1000 PRO microplate reader (TECAN) and the accompanying software Magellan. The 96-well microplates used were purchased from Greiner Bio-one (Cat. No. 655901).

### Measurement of β-glucuronidase Activity

The procedure used for measuring *β*-glucuronidase activities was modified from Miller [35] and Israelsen et al. [36].

### Sequence Analysis

Protein sequences were obtained from UniProt (http://www.uniprot.org/). Nucleotide sequences were downloaded from GenBank (http://www.ncbi.nlm.nih.gov/genbank/). Multiple alignments and phylogenetic construction were performed using MUSCLE [37] in CLC Main Workbench (http://www.clcbio.com/products/clc-main-workbench/). Phylogenies were visualized in FigTree (http://tree.bio.ed.ac.uk/software/figtree/). RNA secondary structure was predicted using Vienna RNA Web Services [38].

## Results

### Homologous sequences of AckA1 and AckA2 in *L. lactis* MG1363

Protein sequences of the two ACKs in MG1363, AckA1 and AckA2 encoded by *ackA1* and *ackA2* respectively, are homologous with an identity of 68%. Alignment to ACKs from *Salmonella typhimurium* and *Methanosarcina thermophila* (*M. thermophila*) whose structures are known [16], [17] (Fig. S1) showed conservation of active site, substrate, nucleotide triphosphate and metal binding sites except one residue in an ATP binding site (V331 of AckA1 and I331 of AckA2 respectively) which is also not conserved among other organisms. It is thus difficult to predict differences in enzymatic properties based on sequences alone.

### Multiple *ackA* genes existing in *L. lactis* and many other species

To understand the evolutionary relationship between the ACKs from *Lactococcus* and other closely related LAB, a search for species with multiple acetate kinases was initiated. Under the genus *Lactococcus*, there are a total of 15 strains of three species, ten *L. lactis*, four *L. garvieae* and one *L. raffinolactis*. Interestingly, all *Lactococcus* strains except *L. raffinolactis* harbor two homologous ACK genes. For *Streptococcus* which is closest to *Lactococcus*, in contrast, among more than 500 strains with available ACK sequences, only 16 of them have two or more ACKs. It is however not a distinctive feature for *Lactococcus* but in fact a very general phenomenon in bacteria. Among the 11,100 entries predicted to encode ACKs in Uniprot, around 5,000 of them are not the unique gene for ACK in an organism. These multiple ACK genes exist in 2,242 strains from 320 species under 135 genera. Interested readers are referred to Table S4 for the complete list and the criteria for distinguishing multiple ACKs.

### Two Types of ACK Conserved in *Lactococcus*

**Figure 2:**
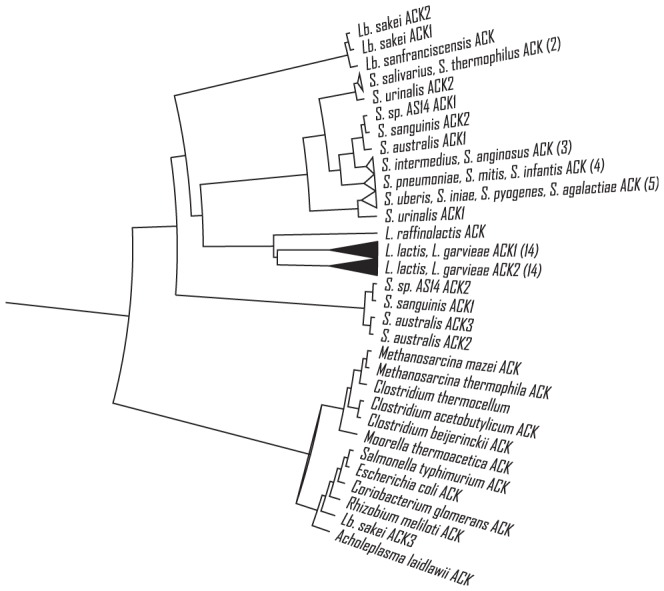
Phylogeny of acetate kinases from *Lactococcus*, *Streptococcus* and other species. One ACK from each *L. lactis* and *L. garvieae* strain forms a monophyletic group and the other ACK forms another (filled triangles). A triangle represents a cluster of sequences lumped in one line and the number in the bracket is the size of the cluster.

A multiple alignment including sequences from all *Lactococcus*, several *Streptococcus* and *Lactobacillus* representatives with the experimentally studied species as outgroup was performed to construct a phylogeny (Fig. 2). From the phylogeny, it became clear that, except for *L. raffinolactis*, one ACK in each *Lactococcus* strain forms a monophyly and the other ACK in each strain forms another (filled triangles in Fig.2). A multiple alignment indicating the conserved differences of amino acid sequences between the two types of ACK in all *Lactococcus* strains is also shown in Fig. S2. For *Streptococcus* and *Lactobacillus*, similar but more complex relationships could be observed. For example, a *S. urinalis* strain has two ACKs more similar to ACKs in other species than to each other. One of the three ACKs of *Lb. sakei* also has a larger divergence with the other two ACKs than with the ACKs from bacteria in other phyla.

From the alignment of the two types of ACK in *L. lactis* with ACKs of known structure [16], [17], [39] (Fig. S2), it was observed that some important residues conserved within each type of ACK were different between the two types, e.g. position 331 (relative to MG1363's AckA1) in an ATP binding site and position 287–291 including a deletion on a helix containing an ATP binding site.

### Predicted Transcription Terminator between the Two ACK Genes in *L. lactis*

**Figure 3:**
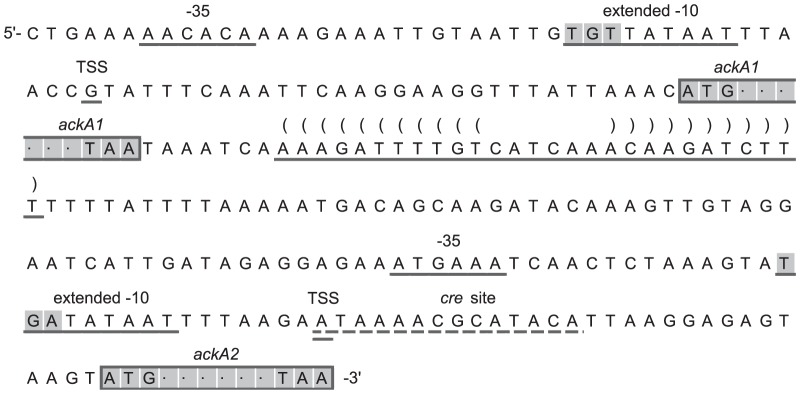
Nucleotide sequence upstream of *ackA1* and *ackA2*. TSS: putative transcription start site. Putative −35 and extended −10 element are underlined. Shaded nucleotides in −10 element: TGn motif. *cre* site responsible for carbon catabolite repression is dotted underscored. Bracket pairs represent the base pairing in the predicted stem-loop structure which is conserved among all *L. lactis* strains.

In all *L. lactis* strains, the genes for the two ACKs are neighbors of each other. In MG1363, the gene upstream was annotated as *ackA1* and the other as *ackA2*. To see if they form an operon, the intergenic RNA secondary structure was predicted using ViennaRNA Web Services [38]. A stem-loop structure followed by a poly-U sequence which is a potential transcription terminator located 8 bp downstream of the stop codon of *ackA1* was predicted (Fig. 3). The prediction is conserved for all sequenced *L. lactis*.

### Distinct Transcription Start Sites for *ackA1* and *ackA2*

A 5′-end RACE was conducted on RNA samples from MG1363 growing on glucose and maltose respectively to locate the transcription start site (TSS) of the two *ackA* genes. For each gene, an individual TSS was identified and putative −35 element and extended −10 element containing a TGn motif [40] were proposed (Fig. 3). We were unable to demonstrate the existence of an additional transcript containing both *ackA2* and *ackA1* although this should have been possible for the RACE approach used.

### Distinct Transcription Units and Activities

**Figure 4:**
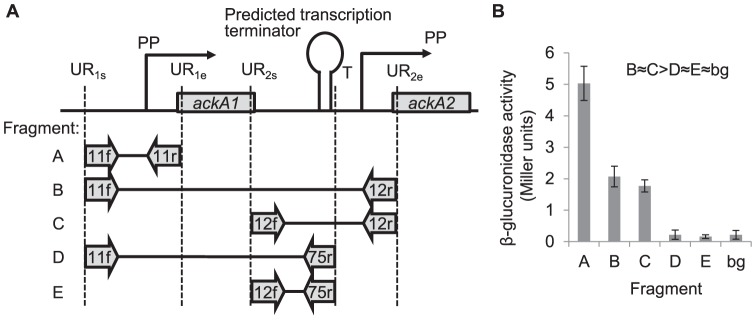
Transcription activities of *ackA* genes. (A) Fragments used for transcriptional fusion. Numbers on arrows refer to primers in Table S1. PP: putative promoter. UR_1s_: ≈500 bp upstream of *ackA1*. UR_1e_: ≈50 bp after the start codon of *ackA1*. UR_2s_: ≈500 bp upstream of *ackA2*. UR_2e_: ≈50 bp after the start codon of *ackA2*. T: position just after the predicted terminator. (B) The β-glucuronidase activities induced by the corresponding fragments. bg: background activity from the control strain without any promoter upstream of *gusA*. Error bars are equal to standard deviations of measurements on three replications.

Reporter fusions were constructed as a quantitative approach to examine the transcription activity of *ackA1* and *ackA2*. Five resulting strains with fragments A–E (Fig. 4a) respectively fused transcriptionally to *gusA* were grown on MSA medium and the *β*-glucuronidase activities were determined (Fig. 4b).

Fragment A including the putative promoter (PP) of *ackA1* resulted in the highest activity of 5 Miller units. Fragment B includes both the PPs of *ackA1* and *ackA2* whereas fragment C includes only the PP of *ackA2*. They resulted in very similar activities (≈2 Miller units). This shows that the PP of *ackA1* had negligible effect on the transcription of *ackA2*. Fragment D and E, starting at the same 5′ end of fragment B and C respectively and both ending just after the predicted terminator but before the PP of *ackA2*, resulted in activities indistinguishable from the background activity (‘bg’ in Fig. 4b). This demonstrates that the predicted terminator is effective and that the transcription of *ackA2* is governed by its own promoter.

### Huge Differences in k_cat_ and K_m_ for acetate

The molecular weights of the His-tagged ACKs were estimated to be 100 kDa for AckA1 and 84 kDa for AckA2 using gel filtration, close to a double of the monomer (43 kDa). Both proteins are concluded to be homodimeric. ACK activities of the enzymes were measured (Fig. S3). Both AckA1 and AckA2 were active in both directions. k_cat_ and K_m_ for all four substrates were estimated (Table 1). Two exceptional differences between AckA1 and AckA2 were first the much higher k_cat_ of AckA1 in both directions (8-fold higher for acetate production and 4-fold higher for the reverse) and second the much lower apparent K_m_ for acetate of AckA2 (1.87 mM) compared to that of AckA1 (22.07 mM).

**Table 1:** Estimated K_m_ and k_cat_ for AckA1 and AckA2.

		AckA1	AckA2
K_m_ (mM)	Ac-P	0.35 (0.02)	0.086 (0.02)
	ADP	0.94 (0.09)	1.15 (0.12)
	OAc	22.07 (1.05)	1.87 (0.29)
	ATP	0.086 (0.0078)	0.21 (0.12)
k_cat_ (s^−1^)	OAc→Ac-P	3234 (221)	394 (27)
	Ac-P→OAc	1033 (98)	282 (28)

Ac-P: acetyl-phosphate. OAc: acetate. Values in bracket represent the standard error of estimation from ≥20 data points.

### Additive Cell Extract Activities of Mutant Strains

**Figure 5:**
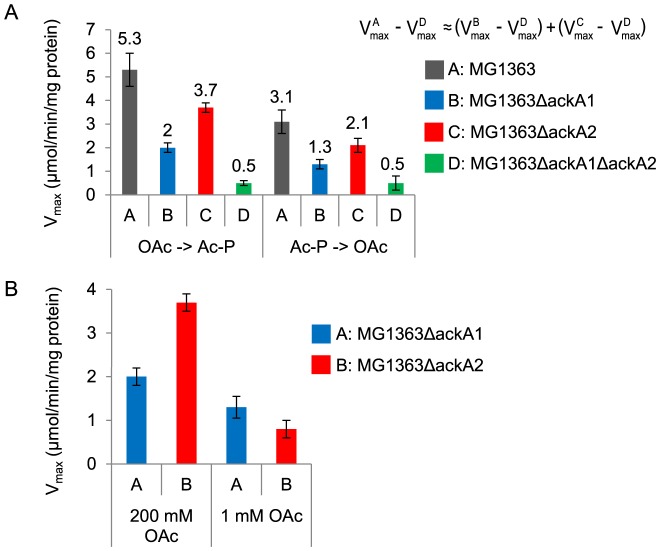
ACK activites in crude extracts of MG1363 and derived *ackA* deletion strains. (A) Activities under normal assay conditions with 200 mM acetate. When subtracting the activity in MG1363Δ*ackA1*Δ*ackA2* (which represents background activity) from the activities in the other three strains, the sum of the activities in MG1363Δ*ackA1* and MG1363Δ*ackA2* was approximately equal to the activity in MG1363. (B) Activities of MG1363Δ*ackA1* and MG1363Δ*ackA2* in the presence of 1 mM or 200 mM of acetate (OAc). Error bars are equal to standard deviations of measurements on three replications.

To study the physiological roles of the two ACKs in *L. lactis*, three mutant strains, MG1363Δ*ackA1*, MG1363Δ*ackA2* and MG1363Δ*ackA1*Δ*ackA2*, were constructed by inactivating *ackA1*, *ackA2* and both respectively. V_max_ of ACK in MG1363 and the three mutant strains growing on MSA media were measured (Fig. 5a). MG1363 showed the highest activity, followed by MG1363Δ*ackA2* and then MG1363Δ*ackA1*. MG1363Δ*ackA1*Δ*ackA2* had the lowest activity. An interesting observation was the additivity of the activities. When subtracting the activity in MG1363Δ*ackA1*Δ*ackA2* (which represents the background activity) from the activities in the other three strains, the sum of the activities in MG1363Δ*ackA1* and MG1363Δ*ackA2* was approximately equal to the activity in MG1363. The implication is discussed below.

To verify the much lower K_m_ for acetate of AckA2, V_max_ was also determined in the presence of 1 mM acetate (Fig. 5b). MG1363Δ*ackA1* did show a higher activity than MG1363Δ*ackA2*. This is opposite to what was observed under normal assay conditions with 200 mM acetate and agrees with the differences in the K_m_.

### Slower Acetate Production by MG1363Δ*ackA1* at a High Extracellular Acetate Concentration

**Figure 6:**
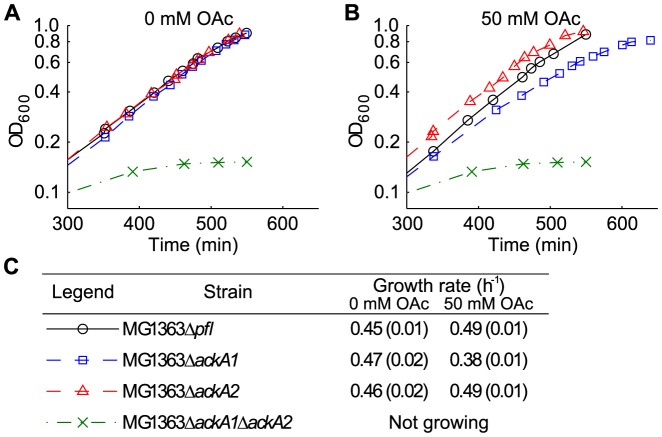
Representative growth curves and growth rates of MG1363 and derived *ackA* deletion strains. Growth with (A) 0 mM and (B) 50 mM acetate. Only data obtained 5 hours after the start of the experiments are plotted for better visualization. (C) Table showing the legend and average growth rates. Values in brackets represent standard deviations of three replications.

To test whether the two ACKs performed differently *in vivo*, growth experiments of MG1363, MG1363Δ*ackA1*, MG1363Δ*ackA2* and MG1363Δ*ackA1*Δ*ackA2* were conducted on MSA media with or without 50 mM acetate. Fig. 6 shows representative growth curves of the four strains and the average growth rates. In all experiments the wild type and single-deletion strains were able to grow up to an OD_600_≈1 where the HPLC analysis showed that the sugar had been consumed (data not shown). The double deletion strain MG1363Δ*ackA1*Δ*ackA2* stopped growing at a much lower cell density of OD_600_≈0.1. When acetate was absent, MG1363 and the two single deletion strains grew with similar growth rates about 0.45 h^−1^. With 50 mM acetate present in the media, MG1363Δ*ackA1* had a significantly reduced growth rate of 0.38 h^−1^, equal to a 20% reduction compared to MG1363 and MG1363Δ*ackA2* (0.49 h^−1^).

The product formation from the three growing strains was also measured (Table 2). In the absence of acetate, the distribution of fermentation products was very similar for all three strains. Since MG1363Δ*ackA1*Δ*ackA2* was unable to grow and produce acetate after OD_600_≈0.1, the acetate production in MG1363Δ*ackA1* and MG1363Δ*ackA2* can be attributed to AckA2 and AckA1 respectively. This implies that both individual ACKs were able to sustain a flux equal to that in the wild type where both ACKs were present. In the presence of 50 mM acetate, however, the reduced acetate production rate concomitant with the reduced growth rate (Fig. 6c), formate production rate and maltose uptake rate in MG1363Δ*ackA1* reveals that the flux entering the mixed acid branch decreased while the lactate flux remained unchanged (Table 2). This indicates that AckA2 alone in MG1363Δ*ackA1* was unable to maintain the same flux as in MG1363 and MG1363Δ*ackA2* where AckA1 was present. It can thus be concluded that AckA1 performed better than AckA2 in acetate production in the presence of a high concentration of acetate (50 mM) in the media.

**Table 2:** Average specific rates of consumption of maltose, production of lactate, formate and acetate of MG1363, MG1363Δ*ackA1* and MG1363Δ*ackA2* at 0 or 50 mM of extracellular acetate.

	Specific rate of consumption/production (mmol h^−1^ gdw^−1^)							
	MSA, 0 mM OAc				MSA, 50 mM OAc			
	maltose	lactate	formate	acetate	maltose	lactate	formate	acetate
MG1363	7.9 (0.5)	20.6 (1.5)	12.5 (0.9)	7.5 (0.8)	8.3 (0.6)	20.1 (1.6)	14.0 (1.4)	7.9 (0.9)
MG1363Δ*ackA1*	8.0 (0.5)	19.9 (1.4)	12.7 (1.0)	8.0 (0.4)	7.2 (0.5)	19.0 (1.8)	8.9 (1.0)	5.1 (1.0)
MG1363Δ*ackA2*	7.3 (0.6)	18.8 (1.1)	12.5 (1.2)	7.4 (0.4)	8.0 (0.6)	18.0 (1.1)	14.3 (1.2)	7.7 (0.7)

OAc: acetate. Values in brackets represent standard deviations of three replications.

### Slower Acetate Uptake by MG1363Δ*ackA2*Δ*pfl* at Low Acetate Concentrations

**Figure 7:**
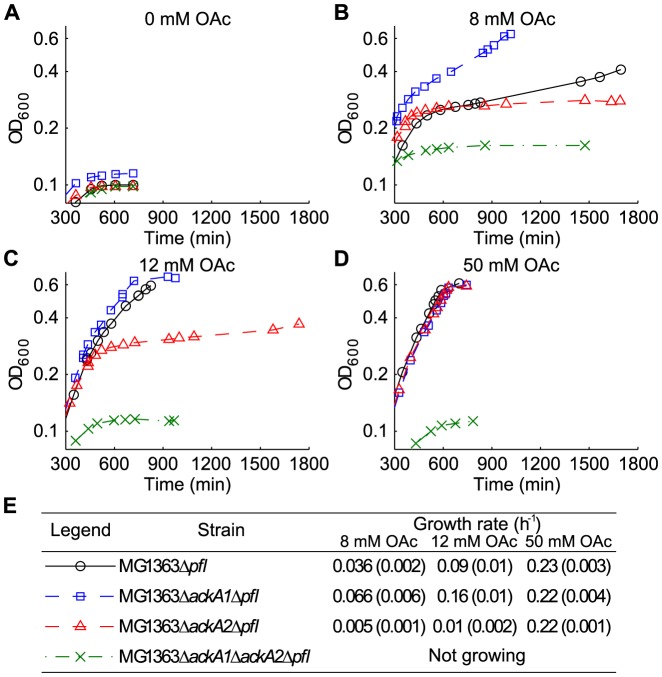
Representative growth curves of MG1363Δ*pfl* and derived *ackA-pfl* deletion strains. Growth with (A) 0 mM, (B) 8 mM, (C) 12 mM and (D) 50 mM acetate. Only data obtained 5 hours after the start of the experiments are plotted for better visualization. (E) Table showing the legend and average growth rates. Values in brackets represent standard deviations of three replications.

An acetate-assimilating growth condition was created by excluding lipoic acid from the medium and knocking out PFL in the *ackA* deletion strains. The PFL-deleted strains, MG1363Δ*pfl*, MG1363Δ*ackA1*Δ*pfl*, MG1363Δ*ackA2*Δ*pfl* and MG1363Δ*ackA1*Δ*ackA2*Δ*pfl*, were grown in MSA media supplemented with acetate. Fig. 7 shows the growth in media supplemented with no acetate, 8 mM, 12 mM and 50 mM acetate respectively. When acetate was absent, all four strains stopped growing at OD_600_≈0.1 (Fig. 7a), showing that the cells depended on acetate for growth beyond this point. When acetate was added to the media, further growth could be seen for all strains except MG1363Δ*ackA1*Δ*ackA2*Δ*pfl*. A clear transition occurred between OD_600_ = 0.2 and 0.3, after which growth depends on acetate. The final OD_600_ was around 0.6 where HPLC analysis indicated that all maltose had been consumed.

Very slow acetate-dependent growth was observed for MG1363Δ*ackA2*Δ*pfl* at 8 or 12 mM of extracellular acetate, >10-fold slower than MG1363Δ*ackA1*Δ*pfl* and MG1363Δ*pfl* (Fig. 7b, c). MG1363Δ*ackA1*Δ*pfl* also grew even faster than MG1363Δ*pfl* at these acetate concentrations. When the acetate concentration increased to 50 mM, however, the growth rates were similar for all three strains (0.22–0.23 h^−1^, Fig. 7d).

The consumption of acetate was also quantified (Table 3). The acetate uptake rate of MG1363Δ*ackA1*Δ*pfl* was significantly higher than that of MG1363Δ*pfl* (2–3 fold) and MG1363Δ*ackA2*Δ*pfl* (>6 fold) at 8 or 12 mM extracellular acetate. They were nonetheless indistinguishable at 50 mM extracellular acetate. Since MG1363Δ*ackA1*Δ*ackA2*Δ*pfl* did not show acetate-dependent growth, the acetate uptake in MG1363Δ*ackA1*Δ*pfl* and MG1363Δ*ackA2*Δ*pfl* could be attributed to the presence of AckA2 and AckA1 respectively. It can thus be concluded that at a high acetate concentration (50 mM), both individual ACKs were able to take up acetate as fast as in MG1363Δ*pfl* whereas at low acetate concentrations (≤12 mM), AckA2 has a significant higher capability for acetate uptake than AckA1.

**Table 3:** Average specific rates of consumption of maltose, acetate and production of lactate of MG1363Δ*pfl*, MG1363Δ*ackA1*Δ*pfl* and MG1363Δ*ackA2*Δ*pfl*.

	Specific consumption/production rate (mmol h^−1^ gdw^−1^)								
	8 mM OAc			12 mM OAc			50 mM OAc		
	maltose	lactate	acetate	maltose	lactate	acetate	maltose	lactate	acetate
MG1363Δ*pfl*	1.3 (0.1)	2.8 (0.20)	0.02 (0.003)	2.7 (0.3)	8.1 (1.0)	0.16 (0.02)	7.1 (0.4)	28 (1)	2.5 (0.5)
MG1363Δ*ackA1*Δ*pfl*	2.1 (0.2)	5.5 (0.7)	0.06 (0.02)	3.7 (0.3)	12.1 (1.0)	0.27 (0.02)	6.8 (0.2)	27 (1)	2.6 (0.4)
MG1363Δ*ackA2*Δ*pfl*	0.2 (0.03)	0.54 (0.09)	N.D.	0.3 (0.05)	0.8 (0.4)	0.04 (0.01)	6.7 (0.3)	27 (1)	2.6 (0.5)

Formate production was not detectable in all cases. Values in brackets represent standard deviations of three replications. N.D.: not detectable.

## Discussion

### Prevalence of multiple ACKs in bacteria

ACK sequence data from Uniprot revealed that the existence of multiple ACKs is a very common phenomenon in bacteria which is interesting and emphasizes its possible importance. Unfortunately, there are few studies on ACK isozymes in the literature except for a spirochete [23]. We believed that the importance of ACK isozymes on acetate metabolism is being neglected while the acetate metabolism in bacteria is still a subject under active research, for instances, current generation of *Geobacter sulfurreducens*'s growth on acetate [41] and acetate dependency of the probiotic *Lactobacillus johnsonii* [42]. Interestingly, the strains used in the two studies also have multiple ACKs. Knowledge on ACK isozymes in these organisms may provide insights into these studies and their applications. The current study attempts to fill the gap by studying the two ACKs in *L. lactis* at different levels.

### *ackA1* and *ackA2* in *L. lactis* probably resulted from gene duplication

Protein sequence analysis revealed the conserved differences between the two ACKs found in *Lactococcus*. This brought an insight into the potential evolutionary advantage of having ACK isozymes. From the simple phylogenetic analysis, the two *ackA* genes may have resulted from a duplication event in a common ancestor of *Lactococcus* which had already been differentiated from *Streptococcus*. To prove this point, however, a more formal phylogenetic analysis is required.

### AckA1 and AckA2 in MG1363 being isozymes rather than subunits

With respect to the expression of *ackA1* and *ackA2*, our results suggest that the two genes are transcribed individually rather than in an operon. This is actually consistent with the Northern Blot results in de Felipe and Gaudu (2009) [43] where a transcript of around 1 kb was found for *ackA1*. Interestingly, in their study, *ackA1* was assumed to encode one subunit of ACK in *L. lactis*. This question may be worth asking because the two ACKs are in fact homologous to each other.

In the current study, nevertheless, the state of being isozymes rather than subunits of one ACK for the two *ackA* gene products was assumed for several reasons. First, we found that the individual His-tagged enzymes could catalyze the reaction in both directions. Second, the *ackA* mutant strains could produce as well as utilize acetate. Third, the crude extracts from *ackA* mutant strains showed additive activities (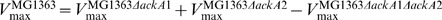). If a hetero-oligomeric form of ACK with different kinetic properties exists, the additivity is less likely to hold. Fourth, most of the ACKs reported in the literature appear to be homodimeric [16], [17], [22], [44]–[47]. Finally, we have data analogous to those in Fig. 4b showing that when growing on glucose, the promoter activity of *ackA2* was 10-fold lower than that of *ackA1* and was close to the value of background activity (unpublished results). This is consistent with the *cre* site identified 6 bp downstream of the *ackA2*'s TSS (Fig. 3) which is subject to carbon catabolite repression [48]. In light of this huge difference between the transcriptional activities, it is unlikely that the two gene products from *ackA1* and *ackA2* form one protein complex. However, the possibility that hetero-oligomeric ACK exists cannot be entirely ruled out.

### Possible different roles suggested by enzyme kinetics

From the His-tagged purified enzymes, AckA1 was shown to have much higher turnover number k_cat_ than AckA2 whereas AckA2 has a higher affinity towards acetate. The apparent K_m_ is the lowest of all the reported ACKs where K_m_ for acetate usually is notoriously high (the highest being 300 mM in *E. coli* [13]). The kinetic properties of the two ACKs thus suggest a possible complementary role in metabolism. For the effect of His-tagging on enzymes, we have looked into the 3D structure of the ACK from *M. thermophile* (PDB ID: 1TUY) [22] which is homologus to AckA1 and AckA2 in *L. lactis*. The first few residues from the N-terminus are outside the catalytic core. Together with the consistency between the assays on purified enzymes and crude extracts, we believed that the His-tag is unlikely to interfere with the reaction.

### Physiological roles of ACK reported in literature

Among the ACKs reported previously, some were found to have ATP production as their primary physiological role while some are more likely to be responsible for acetate activation. For instance, the ACK in *Lactobacillus sanfranciscensis* was suggested to take the role of ATP formation [13]. The ACKs in *Bacillus subtilis* were shown to be non-essential for growth on acetate and meanwhile important for excretion of excess carbohydrate by producing acetate [49].

With respect to examples of acetate activation, in *Corynebacterium glutamicum*, ACK activities were proven to be necessary for growth on acetate [50]. Another interesting case is *M. thermophile* which is acetotrophic. The K_m_ for acetate of the ACK from *M. thermophile* was found to be 22 mM [18]. Site-directed mutagenesis in the same study revealed that only a single-residue mutation could cause a 10-fold lower K_m_ for acetate concomitant with a 6-fold reduction in k_cat_. This striking similarity between the pair of ACKs in *M. thermophile* (wild-type and mutated) and the pair of ACKs in *L. lactis* (AckA1 and AckA2) leaves a possible hint for how AckA1 and AckA2 differentiated and specialized. The author suggested that the sacrifice of a low K_m_ in return for a high k_cat_ conferred the advantage of more rapid acetate uptake to *Methanosarcina* species in an environment with a high acetate concentration [18].

These are only a few examples among many different studies. It must be noted that despite the particular functions of ACK demonstrated in the mentioned studies, one should not exclude other possibilities. The physiological role might be dependent on the nutrients available and complementary to other enzymes like the AMP-forming ACS in bacteria. For example, in *E. coli*, a number of studies on ACK-deficient mutants showed that PTA-ACK is the primary pathway for acetate production, e.g. [51], [52]. Other studies found that it is important for growth on high acetate concentration (≥25 mM) whereas growth on low acetate concentration (≤2.5 mM) depends on ACS [1], [53] (reviewed in [3]). This example of PTA-ACK complementary to ACS in *E. coli* also resembles AckA1 and AckA2 in the sense that ACS has a much lower K_m_ for acetate (0.2 mM) and lower V_max_ [53]. The difference lies in the irreversibility of ACS in *E. coli* and the dependence of AckA2 on PTA to produce Ac-CoA in *L. lactis*. A final example is a spirochete with two ACKs [23]. They had a lower K_m_ for Ac-P and acetate respectively. The authors mentioned the possibility of the two ACKs being specialized in different directions respectively.

### Growth on maltose as a test of the physiological roles in *L. lactis*

To find out whether the two ACKs have different physiological roles, mutant strains were constructed and their response to acetate during growth was examined. In our growth experiments, maltose was chosen as the carbon source because for MG1363 growing on maltose, a more significant amount of formate, acetate and ethanol is produced than on glucose [54]. Via growth on maltose the capacity of the two ACKs to bear a high flux from glycolysis can be tested. Another reason is the much lower ATP/ADP ratio in *L. lactis* when growing on maltose than on glucose. The ATP/ADP ratio in MG1363 was around 9 when growing on glucose [55] and was around 4 when growing on maltose [56]. The lower ATP/ADP ratio provided a more stringent condition for acetate uptake in the mutant strains.

### Complementary roles in acetate metabolism

Our results show that under favorable conditions either one of the ACKs is sufficient for the dual function of acetate production and uptake. In an environment where the concentrations of acetate, lipoic acid (activating PDHc) and oxygen (inactivating PFL) are varying, nonetheless, AckA1 and AckA2 have their own advantages and complement each other to allow fast growth at different extracellular acetate concentrations. In an environment with high acetate concentration (50 mM), AckA1 showed its superior capability of acetate production. This is consistent with the kinetic properties *in vitro*. The much higher affinity for acetate of AckA2 probably led to a larger effect of product inhibition by extracellular acetate diffused into the cells. In contrast, in a dynamic environment where PFL and PDHc are inactive, e.g. containing oxygen and without lipoic acid, our results from the growth experiments of PFL and ACK defective strains show that AckA2 is important for acetate uptake when the acetate source is scarce (≤12 mM). The lower growth yield compared to PFL-effective strains (OD_600_ = 0.6 vs 1) was probably a result of loss of the ATP generated from acetate production combined with additional ATP consumed for acetate uptake.

Another possible function of the two ACKs that should not be overlooked is the emergent properties of combining the isozymes. A possibility is thus a switch to fine-tune the direction and rate of the reaction in response to the cellular requirement by altering the expression of the two *ackA* genes. It would be interesting to look into the regulation of the expression of *ackA1* and *ackA2* to examine this hypothesis.

### PTA-ACK as the only pathway interconverting Ac-CoA and acetate in *L. lactis*

The inability of MG1363Δ*ackA1*Δ*ackA2* and MG1363Δ*ackA1*Δ*ackA2*Δ*pfl* to grow on MSA media suggests the absence of other pathways involved in the interconversion between Ac-CoA and acetate. For MG1363Δ*ackA1*Δ*ackA2*, the only known pathway left for catabolizing Ac-CoA is the NAD^+^-generating bi-functional alcohol dehydrogenase (ADHE) because of the lack of ACKs. The redox imbalance could lead to the accumulation of toxic intermediate metabolites such as acetaldehyde which prevents growth. For MG1363Δ*ackA1*Δ*ackA2*Δ*pfl*, no acetate was assimilated to form Ac-CoA to satisfy anabolic requirements. If other pathways for either direction exist, one of the strains should be able to grow. Thus, these results further emphasize the importance of AckA1 and AckA2 in the acetate metabolism of *L. lactis*. For the initial growth up to OD_600_≈0.1 of the two strains, it was found to be caused by the small amounts of lipoic acid present as impurity in the amino acids composing the media which could activate PDHc. Adding lipoic acid to these cultures indeed allowed growth beyond OD_600_ = 0.1 and growth experiments in media with reduced amounts of amino acids showed that these strains stopped growing at a lower OD_600_ whereas the wild-type MG1363 was unaffected (data not shown). This indicated the presence of small amounts of lipoic acid in the amino acid stock which caused the initial growth of MG1363ΔackA1ΔackA2 and MG1363Δ*ackA1*Δ*ackA2*Δ*pfl*.

### Conclusions

In conclusion, the present study demonstrated the different and yet complementary roles of the two acetate kinases in *L. lactis* MG1363 with one being specialized in acetate production and the other in acetate uptake. It was observed from the sequence and phylogenetic analysis, supported with transcriptional analysis and the enzyme kinetics, and finally confirmed by the different growth behavior of mutant strains harboring only *ackA1* and *ackA2* respectively. The findings can be of great significance in bacterial metabolism in light of the fact that more than 300 species of organisms actually have multiple ACKs. Evolution to multiple ACKs specialized in complementary functions may be a common strategy in bacteria in response to the dual nature of acetate which can be an essential substrate but also an inhibitor for growth depending on environmental conditions.

## Supporting Information

Table S1 **Primers used in this study.**
(DOCX)

Table S2 **Plasmid used in this study.** Erm^r^, Cam^r^ and Amp^r^ stand for erythromycin, chloramphenicol and ampicillin resistance respectively. CDS: coding sequence.
(DOCX)

Table S3 **Strains used in this study.**
(DOCX)

Table S4 **ACK entries in Uniprot.** (a) Complete list downloaded from Uniprot, sorted in alphabetical order of organism names. (b) Identification of multiple ACKs existing in the same organism from the complete list. Criteria to distinguish isozymes from the same strain: 1. Sequence length lies between 350aa and 450aa, where the main cluster of ACK sequences have (≈95%). All reported ACKs in the literature also have length in this range. Ignore other outliers. 2. Exclude entries with the word ‘fragment’ in ‘protein name’ which can mean a fragment of the same protein represented by another entry, leading to false positive. 3. Exclude entries without a specific strain name in ‘organism’, which can be results from metagenomic studies, leading to possible false positive. (c) Entries identified as multiple ACKs existing in the same organism, sorted in alphabetical order of organism names. (d) Counting the number of different strains, species and genera and the number of ACKs in each strain.
(XLSX)

Figure S1 **Multiple alignment of AckA1, AckA2 and ACKs from** ***Salmonella typhimurium*** **and** ***Methanosarcina thermophila*** **.** Structures identified in previous crystallographic studies [16], [17] are annotated.
(EPS)

Figure S2 **Multiple alignment of all lactococcal ACKs.** Residues conserved with each type of ACK but different between the two types are annotated.
(EPS)

Figure S3 **Activities of purified acetate kinases.** (a) AckA1 and (b) AckA2 converting acetate (OAc) into acetyl-phosphate (Ac-P) at different levels of (i) acetate and (ii) ATP and, converting acetyl-phosphate into acetate at different levels of (iii) Ac-P and (iv) ADP, respectively.
(EPS)
