# Supplementary material for: Acetate Kinase Isozymes Confer Robustness in Acetate Metabolism
Source: PLoS One. 2014 Mar 17;9(3):e92256. doi: 10.1371/journal.pone.0092256 (PMC3956926; doi:10.1371/journal.pone.0092256)
Supplement: Table S1 — Primers used in this study. (DOCX) [file pone.0092256.s001.docx]

| Primer | Restriction site | Amplified region/Description | Sequence |
| --- | --- | --- | --- |
| *DNA deletion* | | | |
| 56f | BamHI | *ackA1* core upstream | gacaggatccGGAGGATTTACTGACAAGTG |
| 56r | PstI |  | tactgctgcagCAATGCCGGCAGCATTTG |
| 57f | PstI | *ackA1* core downstream | tactgctgcagCACCTCTTGCTGGAGTG |
| 57r | XhoI |  | gtttactcgagCTAACGTGTTCTTCGTTGTTG |
| 59f | / | Verify *ackA1* core deletion  with 57r | CAAATGCTGCCGGCATTG |
| 60f | BamHI | *ackA2* core upstream | gacaggatccGATGTATGTTGACCGCATTC |
| 60r | XhoI |  | gtttactcgagAAACGTTGGCCGGATTATG |
| 61f | XhoI | *ackA2* core downstream | gtttactcgagGCGATTGAAGGTGGTAAATC |
| 61r | KpnI |  | tagaggtaccCAACAGATCAATTTGCTCATG |
| 63f | / | Verify *ackA2* core deletion  with 61r | CTTTACATAATCCGGCCAAC |
| CSO834 | XbaI | *pfl* upstream | ctagtctagaCAAGTGATGTACCAAATGAC |
| CSO835 | BamHI |  | cgcggatccTTTGAAATCTCCTTTGTTCT |
| CSO836 | BamHI | *pfl* downstream | cgcggatccTTCTTAGTATTAAAAAATATAAAG |
| CSO837 | XhoI |  | ggtactcgagTGTGATTCACCCCTATTTCT |
| CSO852 | / | Verify *pfl* deletion | CTTGAATTCTGTTTGCTATTATC |
| CSO853 | / |  | CTTTGTCAGCATCAATTACTTG |
| *His-tagging* | | | |
| 71f | BglII | *ackA1* gene for His-tagging | actgaagatctACCAAAACATTAGCAGTAAACGCTGGTTCATC |
| 71r | SalI |  | actgagtcgacTTATTTTTTAAGTGCCTCAACGTC |
| 62f | BamHI | *ackA2* gene for His-tagging | gacaggatccGAAAAAACGCTCGCTGTCAAT |
| 62r | SalI |  | tacagtcgacTTATTTAGCCGCTTCGACATC |
| *Construction of gusA reporter strains* | | | |
| 11f | XbaI | 436-bp *ackA1* upstream and  36-bp CDS with a stop codon | atcgatctagaGAGGATTTACTGACAAGTG |
| 11r | PstI |  | atcgactgcagttaTGATGAACCAGCGTTTAC |
| 12f | XbaI | 444-bp *ackA2* upstream and  45-bp CDS with a stop codon | atcgatctagaTGAGATGTATGTTGACCG |
| 12r | PstI |  | atcgactgcagttaTAATGATGAGGAGCCTG |
| 75r | SalI | Anti-sense to *ackA* genes; priming the predicted transcription terminator of *ackA1* and excluding the putative promoter of *ackA2* | actgagtcgacTTCCTACAACTTTGTATCTTGCTGTCAT |
| CSO50 | BamHI | Verify chromosomal integration of pLB85 | ggaaggatccCCCATAGTTCATCAGTTATC |
| CSO263 | / |  | CGCGATCCAGACTGAATG |
| *RACE* | | | |
| 72r | / | 5’-RACE for *ackA1* | CCAGCAAGAGGTGTGAAGCCCAT |
| 73r | / | 5’-RACE for *ackA2* | CGAAAACAGCGACCGCAAGTGCAT |

**Table S1.** Primers used in this study.
