# Supplementary material for: Acetate Kinase Isozymes Confer Robustness in Acetate Metabolism
Source: PLoS One. 2014 Mar 17;9(3):e92256. doi: 10.1371/journal.pone.0092256 (PMC3956926; doi:10.1371/journal.pone.0092256)
Supplement: Table S2 — Plasmid used in this study. Ermr, Camr and Ampr stand for erythromycin, chloramphenicol and ampicillin resistance respectively. CDS: coding sequence. (DOCX) [file pone.0092256.s002.docx]

| Plasmid | Description |
| --- | --- |
| pCS1966 | Contain *oroP* for orotate transporter as counterselection tool for deletion; Erm^r^ [26] |
| pCS1966-*ackA1*core | pCS1966 containing the upstream and downstream regions of the core part of *ackA1* obtained with primer pairs 56-57; Erm^r^ |
| pCS1966-*ackA2*core | pCS1966 containing the upstream and downstream regions of the core part of *ackA2* obtained with primer pairs 60-61; Erm^r^ |
| pCS1966-*pfl* | pCS1966 containing the upstream and downstream regions of *pfl* obtained with primers CSO834-837; Erm^r^ |
| pLB65 | Contain *orf1* expressing phage TP901-1 integrase; Cam^r^ [27] |
| pLB85 | Reporter vector containing *gusA* reporter; Erm^r^, Amp^r^ [27] |
| pLB85-*ackA1* | pLB85 containing fragment A obtained with primer pair 11 (including 36-bp CDS and 436-bp upstream region of *ackA1*); Erm^r^, Amp^r^ |
| pLB85-*ackA12* | pLB85 containing fragment B obtained with primers 11f, 12r (including 36-bp CDS and the promoter region of *ackA2* as well as the CDS and 436-bp upstream region of *ackA1*); Erm^r^, Amp^r^ |
| pLB85-*ackA2* | pLB85 containing fragment C obtained with primer pair 12 (45-bp CDS and 444-bp upstream region of *ackA2*); Erm^r^, Amp^r^ |
| pLB85-*ackA1*term | pLB85 containing fragment D obtained with primers 11f, 75r (fragment B excluding the region immediately after the predicted transcription terminator of *ackA1*); Erm^r^, Amp^r^ |
| pLB85-*ackA2*term | pLB85 containing fragment E obtained with primers 12f, 75r (fragment C excluding the region immediately after the predicted transcription terminator of *ackA1*); Erm^r^, Amp^r^ |
| pQE30 | Commercial vector (Qiagen) used for N-terminal His-tagging; Amp^r^ |
| pQE30-*ackA1* | pQE30 containing the gene *ackA1* amplified using primer pair 71; Amp^r^ |
| pQE30-*ackA2* | pQE30 containing the gene *ackA2* amplified using primer pair 62; Amp^r^ |

**Table** S**2.** Plasmid used in this study. Erm^r^, Cam^r^ and Amp^r^ stand for erythromycin, chloramphenicol and ampicillin resistance respectively. CDS: coding sequence.
