# Supplementary material for: Acetate Kinase Isozymes Confer Robustness in Acetate Metabolism
Source: PLoS One. 2014 Mar 17;9(3):e92256. doi: 10.1371/journal.pone.0092256 (PMC3956926; doi:10.1371/journal.pone.0092256)
Supplement: Table S3 — Strains used in this study. (DOCX) [file pone.0092256.s003.docx]

| Strain | Description |
| --- | --- |
| MG1363 | A plasmid-free strain derived from *L. lactis subsp. cremoris* NCDO 712 [24] |
| MG1363Δ*pfl* | MG1363 with *pfl* deleted using pCS1966-*pfl* |
| MG1363Δ*ackA1* | MG1363 with the core part of *ackA1* deleted using pCS1966-*ackA1*core |
| MG1363Δ*ackA2* | MG1363 with the core part of *ackA2* deleted using pCS1966-*ackA2*core |
| MG1363Δ*ackA12* | MG1363Δ*ackA1* with the core parts of *ackA2* deleted using pCS1966-*ackA1*core |
| MG1363Δ*ackA1*Δ*pfl* | MG1363Δ*ackA1* with *pfl* deleted using pCS1966-*pfl* |
| MG1363Δ*ackA2*Δ*pfl* | MG1363Δ*ackA2* with *pfl* deleted using pCS1966-*pfl* |
| MG1363Δ*ackA12*Δ*pfl* | MG1363Δ*ackA12* with *pfl* deleted using pCS1966-*pfl* |
| LB436 | MG1363 containing pLB65 |
| LB436/blank | LB436 with pLB85 integrated into the TP901-1 attachment site |
| LB436/*ackA1* | LB436 with pLB85-*ackA1* integrated into the TP901-1 attachment site |
| LB436/*ackA12* | LB436 with pLB85-*ackA12* integrated into the TP901-1 attachment site |
| LB436/*ackA2* | LB436 with pLB85-*ackA2* integrated into the TP901-1 attachment site |
| LB436/*ackA1*term | LB436 with pLB85-*ackA1*term integrated into the TP901-1 attachment site |
| LB436/*ackA2*term | LB436 with pLB85-*ackA2*term integrated into the TP901-1 attachment site |
| M15 pREP4 *groESL* | An *E. coli* strain for protein overexpression [25] |
| SC136 | M15 pREP4 groESL containing pQE30-*ackA1* |
| SC137 | M15 pREP4 groESL containing pQE30-*ackA2* |

**Table S3.** Strains used in this study.
